# Influence of tooth loss on mandibular morphology: A cone-beam computed tomography study

**DOI:** 10.4317/jced.55879

**Published:** 2019-09-01

**Authors:** Shaimaa-Mohamed Fouda, Mohammed M. Gad, Maha El Tantawi, Jorma I. Virtanen, Kirsi Sipila, Aune Raustia

**Affiliations:** 1 MSc. Lecturer, Department of Substitutive Dental Sciences, College of Dentistry, Imam Abdulrahman Bin Faisal University, Dammam, Saudi Arabia; 2 Professor, Department of Preventive Dental Sciences, College of Dentistry, Imam Abdulrahman Bin Faisal University, Dammam, Saudi Arabia; 3 PhD. Adjunct Professor, Department of Community Dentistry, Faculty of Medicine, University of Turku, 20014 Turku, Finland; 4 PhD. Medical Research Center Oulu, Oulu University Hospital and University of Oulu, Oulu, Finland; 5 PhD. Professor, Research Unit of Oral Health Sciences, Prosthetic Dentistry and Stomatognathic Physiology, Faculty of Medicine, University of Oulu, P.O. Box 5281, 90014 Oulu, Finland

## Abstract

**Background:**

Tooth loss adversely affects patients’ health and psychosocial wellbeing. In addition, it changes mandibular morphology. Objective: To evaluate the effect of tooth loss, age, and gender on mandibular morphology.

**Material and Methods:**

Cone-beam computed tomographic (CBCT) scans of 101 patients were examined to measure the gonial angle (GA), ramus height (RH) and condylar height (CH). Patients’ age, gender, and dental status were recorded. Repeated measures analysis of variance (ANOVA) was used to assess the impact of gender, age, and tooth loss on the GA, RH and CH. The mean measurements of the GA, RH and CH were compared between dentate/edentulous patients after splitting by gender.

**Results:**

The GA was larger in edentulous patients compared to dentate ones, in females than in males, and in older than in younger. RH on the right side was significantly longer than on the left side (*P*< 0.0001), and also longer in males and younger patients. CH was shorter in younger than in older patients and in dentate than in edentulous patients.

**Conclusions:**

Tooth loss is associated with changes in mandibular morphology and its prevention would avoid these irreversible changes.

** Key words:**Tooth loss, mandibular morphology, Cone-Beam computed tomography, gender, age.

## Introduction

Tooth loss has several drawbacks on quality of life. It has a negative impact on patient’s physical and psychological states (1). Tooth loss may be associated with diabetes, heart diseases and even death (2-4). In addition, tooth loss is also associated with changes in mandibular morphology (5). These changes may affect the structure and function of the masticatory muscles, which in turn influence their proficiency (6). Several studies have investigated the relationship between edentulousness and changes in mandibular morphology (7-12). An increase of the gonial angle (GA) among edentulous patients was reported by some studies, while other studies could not find a significant association (5,7-10). In addition, inconsistent results were noted regarding changes in the gonial angle in relation to age and gender (7,8). Joo et al. (9) found a larger gonial angle in women compared to men, whereas some other studies could not show any association between gender and size of gonial angle (7,10). It has been suggested that changes in the gonial angle is related to older age only if this is associated with tooth loss (11). On the other hand, Fish (12) stated that the gonial angle may be affected by tooth loss and age but they are not the only influential factors. Changes in the ramus height (RH) and condylar height (CH) in relation to tooth loss, age, and gender were investigated previously but conflicting results were reported (5,10).

Panoramic radiographs and lateral cephalograms have been previously used to determine the size of the gonial angle with an equal degree of accuracy (13,14,15). Recently, cone-beam computed tomography (CBCT) has shown better imaging results compared to conventional two-dimensional imaging, allowing more precise measurements (16,17). Studies concerning mandibular morphology among edentulous and dentulous patients are limited, focusing mainly on changes in mandibular morphology among edentulous patients.

The aim of this study was to assess the impact of tooth loss, age, and gender on mandibular morphology in the adult Saudi population. The hypothesis of the study was that age and gender are associated with changes in the morphological features of the mandible that is equal to that of tooth loss.

## Material and Methods

This cross-sectional retrospective study was conducted at the College of Dentistry, Imam Abdulrahman Bin Faisal University, Dammam, Saudi Arabia between the years 2016-2018. The College of Dentistry is the only dental school in the Eastern Province of Saudi Arabia.

All patients who visit the college undergo screening that includes a clinical and radiographic examination before they are referred to the clinics of different specialties to receive treatment. CBCT is not performed on all patients but only in cases when the treatment plan requires further investigations. The study was approved by the Scientific Research Unit at the College of Dentistry, Imam Abdulrahman Bin Faisal University (# 2017019). The patients consented to using their data or radiographs for research purpose.

Patients were included in the study if they fulfilled the following inclusion criteria: 1) had available CBCT scans, 2) had complete dental records and 3) were older than 20 years of age. CBCTs of poor quality were excluded from the study. The number of cases fitting the inclusion criteria and available for analysis in the study was 101. Patients records were reviewed to extract information about age and gender.

The patients were divided according to pattern of tooth loss into: (a) completely edentulous patients and (b) dentate patients, and by age into two groups, under 50 years and 50 years or above. Subjects were considered dentate if at least one intact and / or carious tooth was recorded, others were considered edentulous.

**Figure 1:**
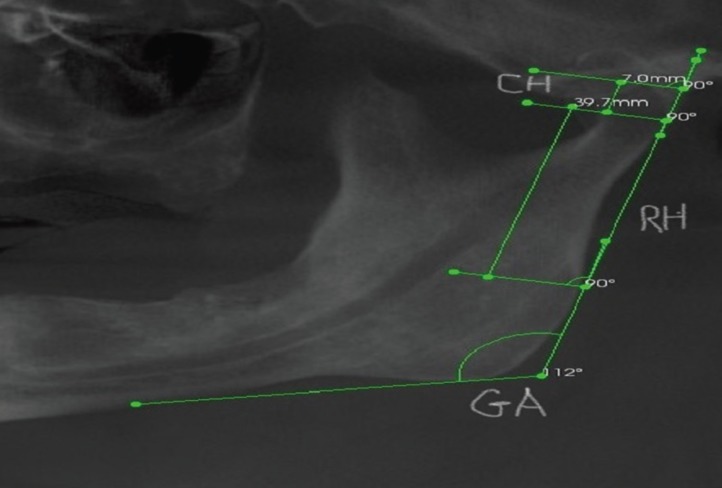
Measurement of gonial angle (GA), ramus height (RH) and condyle height (CH).

-Radiographic examination:

To insure standardization, all included CBCT scans were taken by the same equipment (CS 9300, Carestream) using the same exposure settings. Measurements were taken by the same person using the same software (CS Imaging Patient Browser 7.0.20. Copyright Carestream, Inc.2016). The gonial angle (GA), ramus height (RH) and condylar height (CH) were measured digitally on the panoramic view, according to the methods reported previously (10,13,14) and as shown in Figure 1.

GA was measured by tracing a line tangential to the most inferior points along the lower border of the mandibular body and another line tangential to the posterior borders of the ramus and the condyle and the intersection of these lines formed GA (13,14). RH was measured from the most posterior point of the ramus and CH was measured from ramus height to the most superior point of the condyle (10).

-Statistical Analysis:

Comparison between means of the right and left sides of the GA, RH and CH were done using a t-test. Repeated measures analysis of variance (ANOVA) was used to assess the impact of gender, age (categorized into 2 classes; <50 years and 50+ yrs), and tooth loss (completely edentulous and dentate) on the GA, RH and CH. The mean measurements of the GA, RH and CH were compared between dentate and edentulous patients after splitting by gender. Statistical analysis was done using SPSS version 22.0.

## Results

Of the 101 patients included in the study, 49 (48.5%) were males and 52 (51.5%) were females ( Table 1). Almost half of them (51.5%) were younger than 50 years of age and 13 (12.9%) were edentulous. A greater percentage of males were over 50 years old than females (53.1% and 44.2%) and a greater percentage of females than males were dentate (90.4% and 83.7%).

**Table 1:**
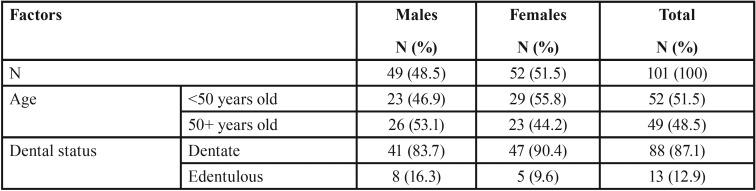
Distribution of the 101 patients examined by cone-beam computed tomography according to age, gender, and dental status.

Comparing the gonial angles (GA) (in degrees) and ramus (RH) and condylar (CH) heights (in mm) of the mandible in 101 subjects measured on CBCT scans, the GA was greater on the left than on the right side (mean= 126.04 and 125.83) and the CH was also greater on the left than on the right side (mean= 7.03 and 8.81). These differences were not statistically significant (*P*= 0.56 and 0.10). On the other hand, the RH was significantly greater on the right side (mean= 39.81) than on the left side (mean= 38.10, *P*< 0.0001) ( Table 2).

**Table 2:**
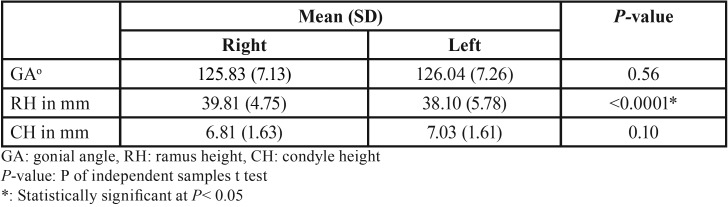
Gonial angles (GA) (in degrees) and ramus (RH) and condylar (CH) heights (in mm) of the mandible in 101 subjects measured on CBCT scans.

In males, the right and left GA, RH, and CH were greater in edentulous patients than in dentate patients and this difference was statistically significant in the case of right and left CH ( Table 3). In females, edentulous patients had a larger right and left GA with no statistically significant differences (*P*= 0.38 and 0.25). The RH and CH were greater in dentate females than in edentulous females and the difference was statistically significant in the case of the RH on the right side (*P*= 0.04) and CH on the left side (*P*= 0.001).

**Table 3:**
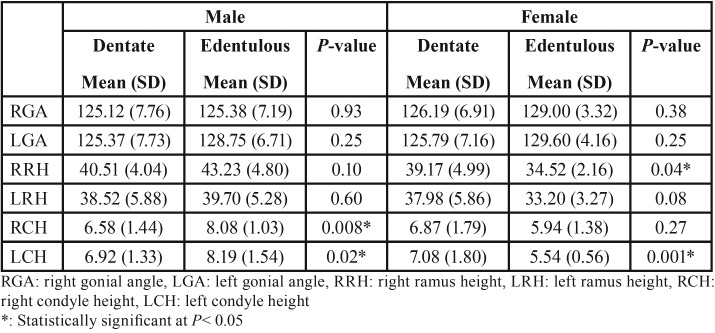
Morphological features of the mandible according to dental status and gender.

 Table 4 shows that males had a smaller GA than females (B= -1.52 and -0.60 on the right and left sides) and so did younger versus older patients (B= -1.95 and -1.88 on the right and left sides) and dentate versus edentulous patients (B= -0.67 and -2.96) although none of these differences were statistically significant.

**Table 4:**
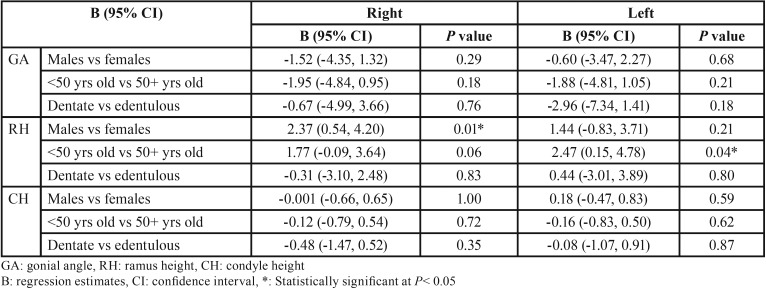
Effect of gender, age and edentulousness on mandibular morphologic features.

The RH was larger in males than females (B= 2.37 and 1.44 on the right and left sides) and in younger versus older patients (B= 1.77 and 2.47 on the right and left sides). These differences were significant between genders on the right side for the RH and by age on the left side (*P*= 0.01and 0.04). The CH was smaller in younger than in older patients on both sides (B= -0.12 and -0.16) and in dentate versus edentulous patients on the right and left sides (B= -0.48 and -0.08) although none of these differences was statistically significant. 

## Discussion

The results of the current study agree with previous studies which reported changes in mandibular morphology following tooth loss (5,10,18). Most of the previous studies investigating changes in mandibular morphology relied on panoramic radiographs for the evaluation of such changes whereas studies using CBCT are more limited (10,15,16). Nowadays, CBCT is being widely used in dental care to overcome the drawbacks of conventional 2-dimensional imaging; the measurements are also more precise because they are performed digitally (17-19). It provides better potential for diagnosis and has better reproducibility compared to conventional 2-dimensional imaging (20).

The consequences of tooth loss on mandibular morphology has been studied before, but contradictory results were reported regarding changes in the GA, RH and CH (5,8-10).

The GA is affected by the strength of contraction of the masticatory muscles (9,21). The function of the masticatory muscles is decreased following partial or complete tooth loss, which in turn may affect the GA and other morphological features (22). In the present study, changes in the GA in relation to the study variables did not show a statistically significant difference, however, a larger gonial angle was found in edentulous patients compared to dentate and in females versus males. Tozoğlu and Çakur (16) reported similar results of a larger GA in edentulous patients although not statistically significant. The results of the present study also are in line with those of previous studies which did not find a significat association between edentulousness and changes in the GA (23,24). However, a significant increase of the GA in edentulous patients compared to dentate patients was reported (5,18).

We found that females had a larger gonial angle than males but the difference was not statistically significant. Huumonen *et al.* (5) found that females had a significantly larger GA than males. However, several studies reported that gender is not associated with changes in the GA (10,15,16).

The mean GA on the left side was slightly larger than on the right side with nonsignificant difference between both sides. This result agrees with the previous studies that reported a nonsignificant difference between right and left GA (15,16,25,26). Similarly, Raustia and Salonen (10) reported a statistically significant smaller right GA among complete denture wearers. Bhardwaj *et al.* (27) and Chole *et al.* (28) found that the values of the left GA is significnalty greater than the right angle. The smaller size of the right GA may be explained by the use of the right side more frequently during mastication (10). On the contrary, Huumonen *et al.* (5) found a larger GA on the right side compared to the left side among dentate women.

A decrease in the RH was reported previously among edentulous patients, which agrees with our results (5). However, some studies reported a larger RH of edentulous mandibles (18,29). A significant difference with a greater RH on the right side was found in our study when comparing the right and left sides. This agrees with a study which reported a significantly greater RH on the right side of edentulous and dentulous women and dentulous men (5).

The present study did not show a significant difference of the CH between both sides. The mean values of the CH showed a statistically significant shorter CH among dentate male patients, however shorter condyles were observed among edentulous female patients with a significant difference on the left side. These results partly agree with Huumonen *et al.* (5) who found a significantly smaller CH among edentulous patients 60 years and older compared to dentate patients. Similarly, Sairam *et al.* (18) found higher values of the CH among old dentulous patients versus the edentulous group. On the other hand some studies did not find a significant correlation between the CH and dental status (9,23).

Our results have implications for rehabilitative patient care to restore function in different age groups. Tooth loss - a modifiable factor- may affect patient appearance by impacting mandibular features in the same way as age, which is an unmodifiable factor. Immediate prosthetic care following tooth loss thus helps in preventing advanced changes that cannot be reversed and may affect patients’ appearance and consequently their psychology.

One of the strengths of our study is the use of CBCT, which improves the precision of measurements. However, because the technique is not universally used for all patients, the sample size we were able to recruit might have posed some limitation especially in reducing the study power for subgroup analysis and reducing the possibility of detecting statistically significant differences.

It can be concluded that tooth loss is associated with changes in mandibular morphology. Prevention of tooth loss is important to avoid irreversible changes in mandibular morphology.
